# Application of an interstitial and biodegradable balloon system for prostate-rectum separation during prostate cancer radiotherapy: a prospective multi-center study

**DOI:** 10.1186/1748-717X-8-96

**Published:** 2013-04-23

**Authors:** Eliahu Gez, Shmuel Cytron, Rahamin Ben Yosef, Daniel London, Benjamin W Corn, Shlomi Alani, Giovanni Scarzello, Fabrizio Dal Moro, Guido Sotti, Filiberto Zattoni, Ike Koziol, Taryn Torre, Matthew Bassignani, Shalom Kalnicki, Reza Ghavamian, Dukagjin Blakaj, Mitchell Anscher, Martin Sommerauer, Dieter Jocham, Corinna Melchert, Stefan Huttenlocher, Gyoergy Kovacs, Madhur Garg

**Affiliations:** 1 Tel Aviv Sourasky Medical Center, Department of Radiation Oncology, 6 Weizmann Street, Tel Aviv, 64239, Israel

**Keywords:** Implantable biodegradable balloon, Prostate-rectum separation, Prostate radiotherapy

## Abstract

**Background and purpose:**

Rectal toxicity presents a significant limiting factor in prostate radiotherapy regimens. This study evaluated the safety and efficacy of an implantable and biodegradable balloon specifically designed to protect rectal tissue during radiotherapy by increasing the prostate–rectum interspace.

**Patients and methods:**

Balloons were transperineally implanted, under transrectal ultrasound guidance, into the prostate–rectum interspace in 27 patients with localized prostate cancer scheduled to undergo radiotherapy. Patients underwent two simulations for radiotherapy planning--the first simulation before implant, and the second simulation seven days post implant. The balloon position, the dimensions of the prostate, and the distance between the prostate and rectum were evaluated by CT/US examinations 1 week after the implant, weekly during the radiotherapy period, and at 3 and 6 months post implant. Dose-volume histograms of pre and post implantation were compared. Adverse events were recorded throughout the study period.

**Results:**

Four of 27 patients were excluded from the evaluation. One was excluded due to a technical failure during implant, and three patients were excluded because the balloon prematurely deflated. The balloon status was evaluated for the duration of the radiotherapy period in 23 patients. With the balloon implant, the distance between the prostate and rectum increased 10-fold, from a mean 0.22 ± 0.2 cm to 2.47 ± 0.47 cm. During the radiotherapy period the balloon length changed from 4.25 ± 0.49 cm to 3.81 ± 0.84 cm and the balloon height from 1.86 ± 0.24 cm to 1.67 ± 0.22 cm. But the prostate-rectum interspace distance remained constant from beginning to end of radiotherapy: 2.47 ± 0.47 cm and 2.41 ± 0.43 cm, respectively. A significant mean reduction in calculated rectal radiation exposure was achieved. The implant procedure was well tolerated. The adverse events included mild pain at the perineal skin and in the anus. Three patients experienced acute urinary retention which resolved in a few hours following conservative treatment. No infections or thromboembolic events occurred during the implant procedure or during the radiotherapy period.

**Conclusion:**

The transperineal implantation of the biodegradable balloon in patients scheduled to receive radiotherapy was safe and achieved a significant and constant gap between the prostate and rectum. This separation resulted in an important reduction in the rectal radiation dose. A prospective study to evaluate the acute and late rectal toxicity is needed.

## Introduction

Prospective randomized trials have demonstrated the advantage of dose-escalated radiotherapy in the treatment of localized prostate cancer [1-3]. Despite the implementation of new radiotherapy technologies, such as intensity modulated radiation therapy and image guided radiation therapy, rectal toxicity has remained high, thus limiting dose escalation [4-8]. Increased separation between the rectum and prostate gland is expected to reduce the rectal dose and improve both radiotherapy safety and efficacy [9]. Two clinically relevant methods have been tested to achieve this goal. The first involves the injection of hyaluronic acid and human collagen to create a space between the prostate and rectum. The approach with hyaluronic acid was tested and a significant stable space between the prostate gland and the rectum was achieved. Although the group of patients is small, the results are promising and require further research [10-12]. The second method involves the insertion of an intra-rectal balloon to separate the rectum from the prostate. With the exception of the anterior rectal wall, this technique reduces the rectal dose, and achieves further stabilization of the prostate. The intra-rectal balloon in daily practice appears to achieve satisfactory results [13].

BioProtect Ltd, Israel has developed ProSpace™, a biodegradable balloon designed for transperineal implantation between the prostate and rectum prior to external beam prostate radiotherapy. The balloon has been shown to remain inflated during the entire radiotherapy period, and then later biodegrade without toxicity [14]. The balloon’s safety and efficacy in separating the anterior rectal wall from the surrounding tissue was established in a mammalian model [15].

This prospective, international multi-center study evaluated the safety of the implant procedure of a biodegradable balloon (ProSpace™) and its efficacy in creating and maintaining a significant space between the rectum and prostate during the radiotherapy period.

### Patients and methods

The study included 6 medical centers with different radiation techniques and schedules (IMRT and 3-DCRT). Because the aim of this study was the safety of the balloon implant procedure and the ability to create a predictable and consistent space between the prostate and rectum that was maintained during the entire period of radiotherapy, the variations in treatment technique and schedule were acceptable. This study was approved by local institutional review boards and all patients participating in the study signed the approved informed consent form (NIH registration number NCT00462124).

Patients: Patients with localized prostate cancer (T1-2, N0, and M0) and 0–1 performance status scheduled for prostate external beam radiotherapy were eligible for this study. Normal blood counts, biochemistry, and clotting test results, peak flow rate >13 ml/sec, and residual urine volume below 150 mL were required at baseline. Patients with a history of prior pelvic radiotherapy, prostatectomy, cryosurgery or other surgical procedures involving the prostate or peri-rectal and peri-prostatic areas were excluded from the study. The implant procedures were performed under general anesthesia; therefore patients with unstable angina pectoris, uncontrolled congestive heart failure or recent myocardial infarction were ineligible for enrollment in the study.

Methods*:* ProSpace™ contains an introducer and a triangular-shaped balloon made of poly (L-Lactide-co-caprolactone) which is a co-polymer of Poly Lactide acid and epsilon Caprolactone, in a ratio of 70:30, a widely used, medically biodegradable material. To enable insertion, the balloon was folded into a cylindrical insertion tube. The balloon was implanted in the Denonvilliers’ fascia, transperineally and guided by transrectal ultrasound (TRUS). After positioning the insertion tube spacer between the prostate and the rectum, the balloon was inflated with sterile saline**.** The implant procedures were performed with the patient under either general or local anesthesia.

All patients underwent two CT-based simulations for radiotherapy treatment planning--the first before the implant, and a second CT simulation one week after implant. Radiotherapy delivery was based on the second simulation and treatment plan. The Clinical Target Volume (CTV) was defined as the prostate gland and seminal vesicles. The Planning Target Volume (PTV) included the CTV with a 5 mm margin at the posterior border, and a 10 mm margin everywhere else. The external surface of the entire urinary bladder, the entire rectum (from the anal verge to the recto-sigmoid) and both hip joints were contoured. The radiotherapy program was according to the local policy. During weekly radiotherapy sessions, and at follow-up visits 3 and 6 months post implantation, a cone-beam CT or ultrasound examination was performed to verify the position, geometry, and integrity of the balloon.

Dose-volume histograms (DVH) of pre and post implant treatment plans were compared to evaluate the impact of the balloon implant on the exposure of the rectum to radiation. Although there is no consensus concerning the rectal dose constraint, the V60 (volume receiving ≥60 Gy) has consistently demonstrated an association with a risk of Grade ≥2 rectal toxicity or rectal bleeding. Based on this study and others, the following dosimetry criteria were chosen for evaluation: V50, V60, D50, D70, D80, D90 and D100 [16-19].

Target volumes were localized weekly with several methods as a function of institutional preference (e.g., cone beam CT, ultrasound). The device was particularly easy to visualize with the CBCT technology. For the purpose of this study and independent review, all image data was transferred to an independent imaging specialist who evaluated the following parameters: distance between prostate and rectum, and the dimensions of the implantable balloon and prostate gland. These parameters were measured and analyzed before and after the implant procedure, weekly during the radiotherapy period, and at 3 and 6 months post-implant.

Adverse events (AE) observed or reported during balloon implantation, throughout the radiotherapy period, and for up to 6 months post implantation, were evaluated according to the Common Terminology Criteria for Adverse Events (CTCAE) Version 3.0 [20]. Pain was scored using the visual analog scale (VAS).

Statistical analyses: Data were analyzed using SAS® V9.2 (SAS Institute, Cary NC, USA) and are presented in tabular format. Continuous variables are summarized by a mean ± standard deviation (or range) and categorical variables by a count and percentage. The calculated differences between pre-balloon and post-balloon rectal dosimetry values were assessed using the Wilcoxon Signed Rank test. A p-value ≤0.05 was considered statistically significant.

## Results

Patients: From June 19, 2009 through November 23, 2010, 27 patients with localized prostate cancer from six medical centers were enrolled in this study. The radiation doses and the delivery techniques were in accordance with local policies. Total radiation dose ranged from 70 Gy to 78 Gy with a daily fraction of 1.8, 2.0 and 2.5 Gy. Two of the centers used conventional 3-dimensional conformal radiation therapy and four centers utilized intensity modulated radiation therapy (Table 1). Median time from implant to first day of radiotherapy was 17 days and median duration of radiotherapy was 39 days (range 28–45). 27 patients enrolled in the study and were evaluated for the implant procedure. Of these, 23 were evaluated for safety and efficacy of the balloon during the period of radiotherapy. One patient was excluded from this analysis due to an error in the implant procedure, and 3 other patients were excluded due to premature balloon deflation which is believed to be related to transrectal insertion of fiducial markers into the prostate prior to the balloon implant.

**Table 1:** Patient distribution and treatment regimens at participating medical centers

**Medical Center**	**Patients number (%)**	**Radiation dose (Gy)**	**Radiation delivery technique**
Sourasky Medical Center, Israel	8 (30)	70.0 (2.5 X 28)	IMRT
Lübeck University, Germany	2 (7)	72.0 (2.0 X 36)	3DCRT
Padova Medical Center, Italy	9 (33)	78.0 (2.0 X 39)	3DCRT
Massey Medical Center, USA	1 (4)	78.0 (2.0 X 39)	IMRT
Montefiore Medical Center, USA	2 (7)	76.0 (1.8 X 42)	IMRT
Virginia Urology, Richmond, USA	5 (19)	75.6 (1.8 X 42)	IMRT
		78.0 (2.0 X 39)	

Balloon implant procedure: The balloon implant procedure was successful in 26 of 27 patients. In one patient, balloon inflation failed due to a technical error. In the remaining 26 patients, the transperineal implant procedure of the balloon was well tolerated and without complications. There were no episodes of infection or thrombosis. The side effects included (Table 2): pain in the perineal scar (range from 1–7, according to VAS), dysuria grade 1 & 2 and one case of penile bleeding. Three patients developed acute urinary retention and required urinary bladder catheterization which resolved within a few hours. In the single patient for whom the implant failed, the removal of the balloon was without complication.

**Table 2:** Adverse events during the balloon implantation and radiotherapy period

	**Balloon implant procedure**	**Radiotherapy period**
	**Number of patients (%)**	
Number of patients evaluated:	26	23
Pain at the perineal skin (1 & 7 VAS score):	7 (27)	— — — — — — —
Acute pain in the anus (2 & 9 VAS score):	4 (15)	— — — — — — —
Acute urinary retention:	3 (12)	1 (4)
Dysuria and Nocturia (grade 1–2):	3(12)	15 (65)
Penile bleeding:	1 (4)	— — — — — — —
Proctitis (Grade 1):	— — — — — — —	2 (8)
Diarrhea (Grade 1):	— — — — — — —	4 (17)
Signs of blood in feces (Grade 1):	— — — — — — —	1 (4)
Constipation (Grade 1):	— — — — — — —	1 (4)
Erectile dysfunction:	— — — — — — —	1 (4)
Pruritus:	— — — — — — —	1 (4)
Fatigue	— — — — — — —	1 (4)
Decreased urine flow:	— — — — — — —	1 (4)

During radiotherapy the most frequent side effect was dysuria grade 1–2 (58%) and one patient developed acute urinary retention and required urinary bladder catheterization during the radiotherapy period. Two patients had mild proctitis. Other side effects are presented in Table 2.

Balloon status (Table 3): The balloon status for the duration of the radiotherapy period was evaluated in 23 of 26 patients. In all 23 patients, the balloon remained inflated during the entire period of radiotherapy and did not change its position in relation to the prostate gland. At 3 months post implantation, balloon deflation was observed in 8 patients (4 complete and 4 partially) and by 6 months all balloons were completely deflated in all patients. At 6 months balloons were fully biodegraded in all but two patients.

**Table 3:** Balloon status during radiotherapy and throughout follow-up

	**1 week after implantation**	**During radiotherapy**	**3 months post implant**	**6 months post implant**
	**Number of patients**			
Patients evaluated:	**26**	**23**	**23**	**23**
Balloon in place:	**26**	**23**	**23**	**2**
Balloon fully inflated:	**26**	**23**	**15**	**0**
Balloon partly inflated:	**— — —**	**— — —**	**4**	**0**
Balloon deflated:	**— — —**	**— — —**	**4**	**23**

**Figure 1:**
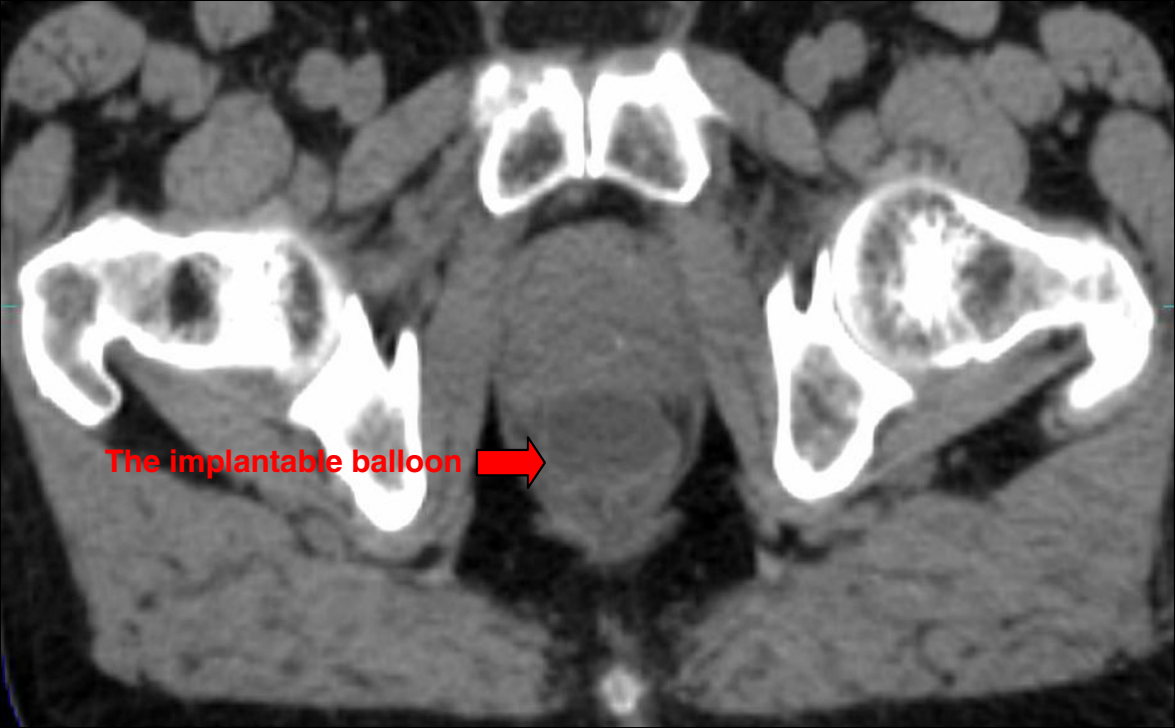
Axial view of CT scan, 7 days post balloon implant.

**Figure 2:**
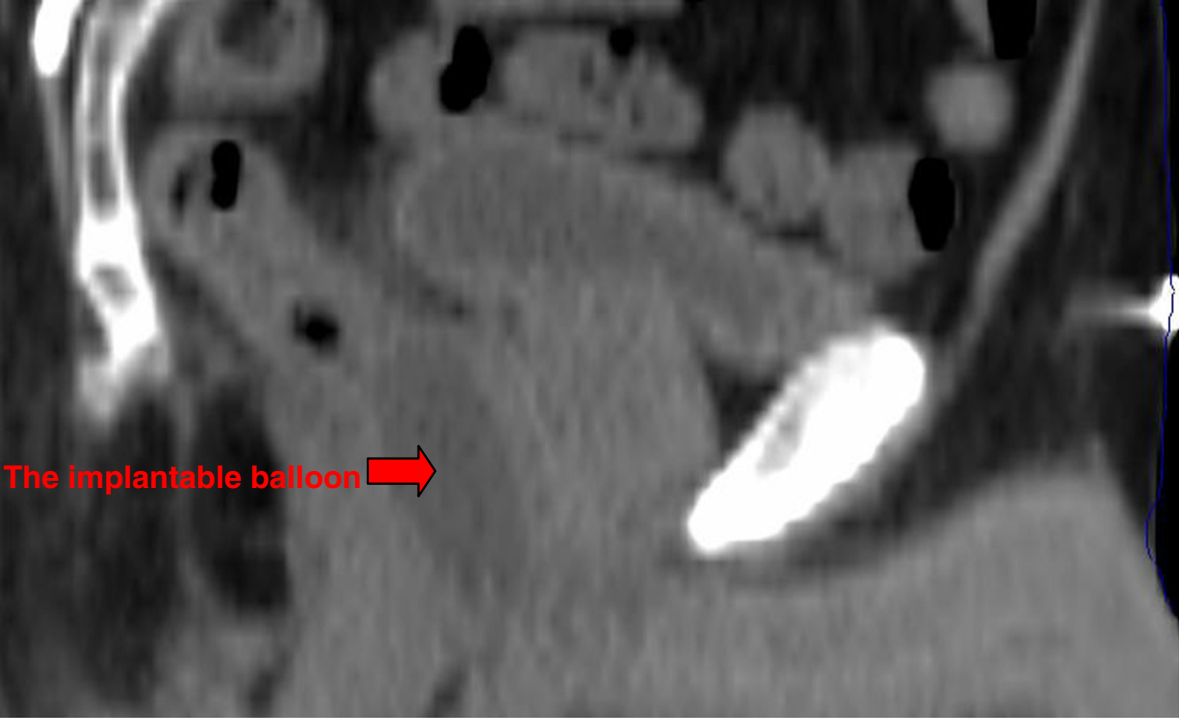
Sagittal view of CT scan, 7 days post balloon implant.

Geometric analysis: The average prostate-rectum distance increased from 0.22 ± 0.2 cm before implant to 2.47 ± 0.47 cm after the implant. The average distance reduced to 2.41 ± 0.43 cm at the end of radiotherapy (statistically non significant). The balloon dimensions 7 days post-implant and at the end of radiotherapy were as follows: width 3.06 ± 0.27 cm and 2.96 ± 0.25 cm (p = 0.03); length 4.25 ± 0.49 cm and 3.81 ± 0.84 cm (p = 0.023); height 1.86 ± 0.24 cm and 1.67 ± 0.22 cm, respectively. Sagittal reconstruction was possible in only 18 of 23 patients. In the remaining 5 patients the reconstruction failed due to the CT scan slice thicknesses that ranged between 2.5 and 10 mm. The three dimensional parameters of the prostate gland before the balloon implant, during radiotherapy period, and at 3 months follow up, did not change significantly (Table 4). Figures 1–2 present axial and sagittal CT scan images demonstrating the location and configuration of the implanted balloon.

**Table 4:** Measurement of the prostate gland, balloon and prostate-rectum interspace

	**Pre implantation**	**Post implantation**	**End radiotherapy**	**P value**	**3-month follow up**	**P value**
# of patients	26	26	18		18	
	Prostate measurements (cm)					
Width	4.37 ± 0.59	4.39 ± 0.64	4.44 ± 0.86		4.15 ± 0.75	
Length	3.74 ± 0.84	4.15 ± 1.08	4.29 ± 0.95		4.10 ± 1.27	
Height	3.42 ± 0.79	3.17 ± 0.61	3.19 ± 0.69		3.14 ± 0.73	
	Balloon measurements (cm)					
Width		3.06 ± 0.27	2.96 ± 0.25	NS	2.62 ± 0.58	
Length		4.25 ± 0.49	3.81 ± 0.84	0.023 *^1^	2.97 ± 1.25	0.05 *^3^
Height		1.86 ± 0.24	1.67 ± 0.22	0.03 *^2^	1.28 ± 0.47	0.003*^4^
	Prostate-rectum distance (cm)					
	0.22 ± 0.2	2.47 ± 0.47	2.41 ± 0.43	NS *^5^	1.59 ± 0.60	0.005*^6^

P values:

The change in length and height of the balloon between post implant and end of radiotherapy, *^1^ & *^2^, respectively.

The change in length and height of the balloon between end of radiotherapy and at 3 months, *^3^ & *^4^, respectively.

The change in the prostrate-rectum distance between post-implant and at the end of radiotherapy; and between the end of radiotherapy and at 3 months post-implant,*^5^ &^*6^.

**Figure 3:**
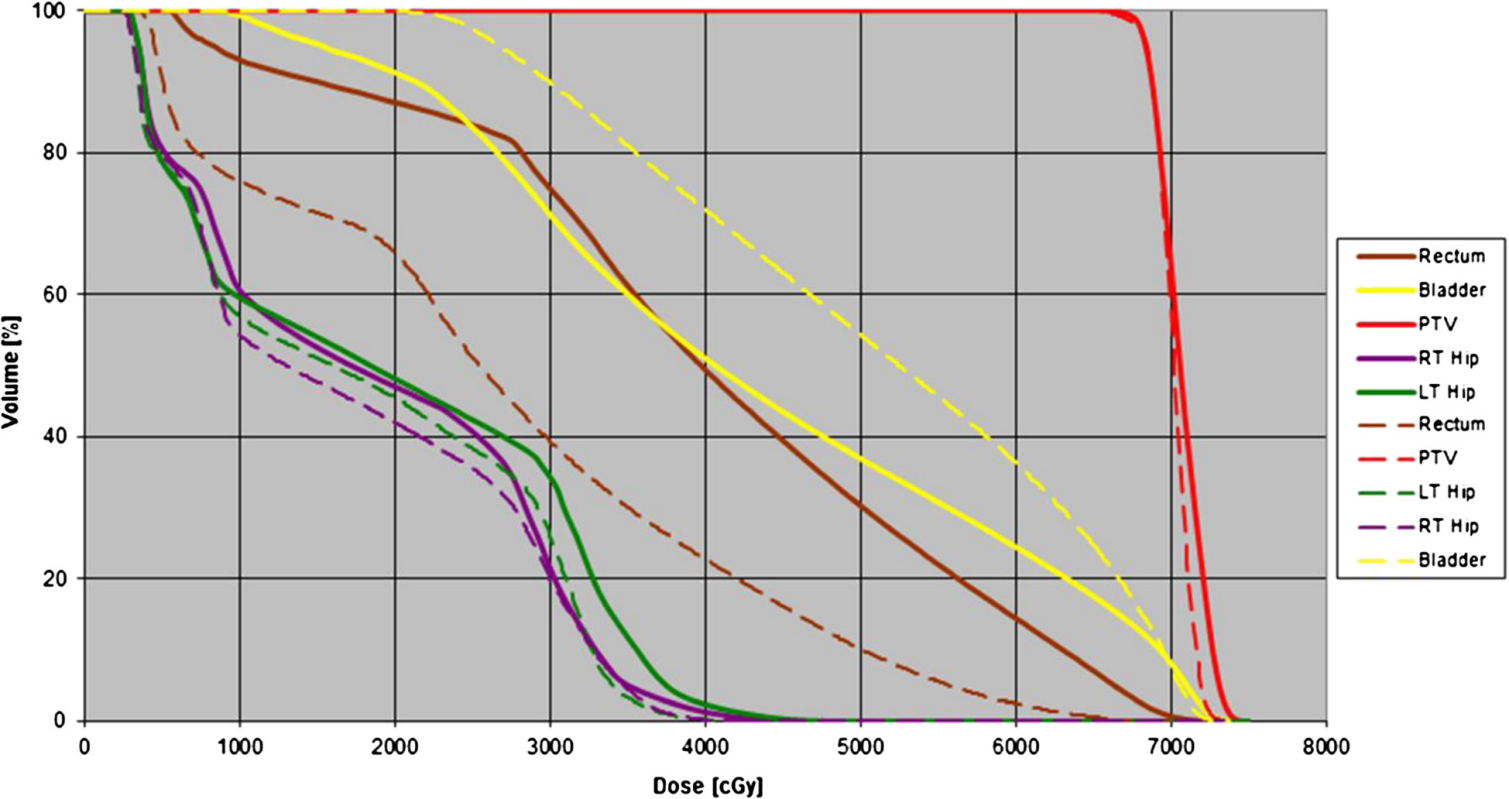
DVHs pre and post balloon implant of a single patient: Continuous line pre and dashed line post balloon implant.

Dosimetry analysis (Table 5): The comparison of the rectal DVH histogram with and without the balloon implant showed a significant decrease in all dosimetry parameters for the rectum*.* Due to the small number of patients that participated, no statistical significance could be expected by evaluating differences in rectal DVH between 3D-CRT and IMRT, and was therefore not compared. A DVH of the PTV and rectum before and after the balloon implant is shown in Figure 3.

**Table 5:** Mean value of rectal dosimetry before and after balloon implant

	**Pre implant**	**Post implant**	**Reduction %**	**P-value**
	**Mean value (Standard deviation)**			
V50	40% ± 17.8	25% ± 17.7	43 ± 28.3	P < 0.0001
V60	30% ± 17.4	15% ± 13.1	57 ± 28.4	P < 0.0001
D50	63Gy ± 20.2	48Gy ± 25.1	25 ± 28.2	P < 0.0001
D70	38Gy ± 16.1	22Gy ± 15.0	46 ± 26.2	P < 0.0001
D80	31Gy ± 15.2	15Gy ± 11.8	57 ± 25.9	P < 0.0001
D90	22Gy ± 14.3	8Gy ± 8.2	67 ± 25.6	P < 0.0001
D100	8Gy ± 8.3	1Gy ± 1.6	82 ± 22.1	P < 0.0001

## Discussion

Rectal toxicity remains a challenging issue in patients receiving radiation therapy for prostate cancer, and as mentioned in the introduction, two different methods have been previously tested to address this problem**.** One is the injection of a hydrogel spacer between the prostate and rectum and the second is the use of an intra-rectal balloon. The aim of this study was to evaluate the efficacy and safety of a new biodegradable balloon transperineally implanted into the rectal-prostate interspace. This international study is based on results from a small group of 27 patients. The implant procedure of the biodegradable balloon was safely performed and was without significant side effects in 26 patients (92%). There was no rectal injury or bleeding. The most frequently reported side effect was dysuria and 3 patients experienced transient acute urinary retention which resolved following conservative treatment and may have been triggered by the use of general anesthesia [21]. In three patients that underwent transrectal insertion of a fiducial marker into the prostate prior to the balloon implant, premature deflation of the balloon occurred before the onset of radiation therapy. We hypothesize that the fiducial markers, inserted through the transrectal approach, remain in a vertical orientation and thereby punctured the balloon resulting in its deflation. To mitigate this problem, fiducial markers were inserted transperineally in the remaining patients. In the remaining 23 patients the biodegradable implantable balloon achieved a significant separation between prostate and rectum. The mean prostate-rectum distance increased from 0.22 ± 0.2 cm to 2.47 ± 0.47 cm after the implant. This distance remained without significant change during the entire period of radiotherapy. The dimension of the implantable balloon decreased slightly during the period of radiotherapy but this change did not affect the prostate-rectum separation. The dosimetry study proved the efficacy of the implantable balloon to decrease the exposure of the rectum to radiation during external beam radiotherapy for prostate cancer. There was a significant reduction in rectal dose and volume parameters. The follow-ups 3 and 6 months post-implant demonstrated the complete deflation of the balloon and its biodegradable property. Preliminary results were presented at ASTRO 2010 and ESTRO 2011.

Our approach is similar in principle to the hydroluronic acid injection concept. Though the materials differ, both methods increase separation between the prostate and the rectum. In both methods, significant and stable spacing was achieved between the prostate and rectum during entire period of radiation therapy. This resulted in a reduction of radiation exposure to the rectum during the treatment of prostate cancer. Our study differs from the intra-rectal balloon method. In our study, the entire rectum is separated from the prostate. In the latter, the anterior wall of the rectum remains proximal to the prostate, and continues to be exposed to a higher dose of radiation. Fixation using the intra-rectal balloon comes at the price of daily insertion which is time-consuming and may be uncomfortable for the patient. With further follow-up, the community of radiation oncologists specializing in treating prostate cancer along with prostate cancer patients will effectively judge which option is most viable.

## Conclusion

Transperineal implantation of the biodegradable balloon (ProSpace™) in patients undergoing prostate radiotherapy is safe and feasible. The balloon is stable and provides a significant gap between prostate and rectal tissues.

A prospective phase II study is needed to confirm the benefits of this implantable balloon on reducing rectal toxicity during external beam radiotherapy of the prostate.

## Competing interests

Rahamim Ben Yosef, MD, Benjamin W Corn, MD and Gyoergy Kovács, MD are consultants for BioProtect Ltd.

## Authors’ contributions

EG principal investigation, planning and delivery radiation therapy and writing the manuscript; SC instructed the method of BioProtect balloon implantation, performed the pilot study and carried out the implantation procedure; RBY study design and review the manuscript; DL radiology evaluation (centralized); BWC draft and manuscript review; SA radiation therapy planning; GS planning and delivery radiation therapy; FDM implantation procedure; GS planning & delivery radiation therapy and patients follow-up; FZ implantation procedure, IK implantation procedure; TT implantation procedure; MB implantation procedure; SK planning and delivery radiation therapy; RG implantation procedure; DB radiation therapy planning; MA patients enrollment, data collection, review data analysis and manuscript, MS implantation procedure; DJ study design and implantation procedure; CM radiation therapy planning; SH radiation therapy planning, GK study design, implantation procedure and review draft and manuscript; MG planning & delivery radiation therapy and review draft and manuscript. All authors read and approved the final manuscript.
